# Could Aspirin and Diets High in Fiber Act Synergistically to Reduce the Risk of Colon Cancer in Humans?

**DOI:** 10.3390/ijms19010166

**Published:** 2018-01-06

**Authors:** Pan Pan, Yi-Wen Huang, Kiyoko Oshima, Martha Yearsley, Jianying Zhang, Jianhua Yu, Mark Arnold, Li-Shu Wang

**Affiliations:** 1 Division of Hematology and Oncology, Department of Medicine, Medical College of Wisconsin, 8701 Watertown Plank Rd, Milwaukee, WI 53226, USA; ppan@mcw.edu; 2 Department of Obstetrics and Gynecology, Medical College of Wisconsin, Milwaukee, WI 53226, USA; ywhuang@mcw.edu; 3 Department of Pathology, John Hopkins University, Baltimore, MD 21218, USA; koshima3@jhmi.edu; 4 Department of Pathology, The Ohio State University, Columbus, Ohio 43210, USA; martha.yearsley@osumc.edu; 5 Center for Biostatistics, The Ohio State University, Columbus, Ohio 43210, USA; jianying.zhang@osumc.edu; 6 Division of Hematology, Department of Internal Medicine, College of Medicine, Comprehensive Cancer Center and The James Cancer Hospital, The Ohio State University, Columbus, OH 43210, USA; Jianhua.yu@osumc.edu; 7 Department of Surgery, The Ohio State University, Columbus, OH 43210, USA; arnold.16@osu.edu

**Keywords:** human clinical trials, aspirin, salicylic acid, cyclooxygenase 2, fruits and vegetables, phytochemicals, synergy, bell/U-shaped, cancer prevention

## Abstract

Early inhibition of inflammation suppresses the carcinogenic process. Aspirin is the most commonly used non-steroid anti-inflammatory drugs (NSAIDs), and it irreversibly inhibits cyclooxygenase-1 and -2 (COX1, COX2). Multiple randomized clinical trials have demonstrated that aspirin offers substantial protection from colon cancer mortality. The lower aspirin doses causing only minimal gastrointestinal disturbance, ideal for long-term use, can achieve only partial and transitory inhibition of COX2. Aspirin’s principal metabolite, salicylic acid, is also found in fruits and vegetables that inhibit COX2. Other phytochemicals such as curcumin, resveratrol, and anthocyanins also inhibit COX2. Such dietary components are good candidates for combination with aspirin because they have little or no toxicity. However, obstacles to using phytochemicals for chemoprevention, including bioavailability and translational potential, must be resolved. The bell/U-shaped dose–response curves seen with vitamin D and resveratrol might apply to other phytochemicals, shedding doubt on ‘more is better’. Solutions include: (1) using special delivery systems (e.g., nanoparticles) to retain phytochemicals; (2) developing robust pharmacodynamic biomarkers to determine efficacy in humans; and (3) selecting pharmacokinetic doses relevant to humans when performing preclinical experiments. The combination of aspirin and phytochemicals is an attractive low-cost and low-toxicity approach to colon cancer prevention that warrants testing, particularly in high-risk individuals.

## 1. Introduction

Analysis of data from eight randomized trials (25,570 patients, 674 cancer deaths) that examined the effects of daily aspirin use on the long-term risk of death due to cancer concluded that daily aspirin use reduced deaths from several common cancers during and after the trials [1]. Benefits increased with the duration of aspirin treatment, as they appeared after five years and lowered the 20-year risk of death from colon cancer. This result was consistent across the different study populations [1,2].

Aspirin was introduced into clinical practice more than 100 years ago, and it belongs to a family of compounds called salicylates, the simplest of which is salicylic acid, aspirin’s principal metabolite. [3]. Salicylic acid is responsible for aspirin’s anti-inflammatory action, as it targets cyclooxygenases-1 and -2 (COX1 and COX2) [4]. Salicylic acid and other salicylates also occur widely in fruits and other parts of plants [5]. Consequently, serum salicylic acid concentrations are greater in vegetarians than in non-vegetarians, and concentrations overlap between vegetarians and people taking low-dose aspirin (≤150 mg per day) [6,7]. Accordingly, aspirin and dietary plants are likely to have synergistic anti-cancer effects. 

In plants, salicylic acid functions as a hormonal mediator of the systemic acquired resistance response to pathogens and environmental stress [5]. Its levels are particularly high in berries, apples, oranges, strawberries, currants, raisins, cucumbers, and tomatoes [5,8]. Some herbs and spices—such as cinnamon, curry powder, rosemary, paprika (hot powder), thyme, and oregano—also contain large amounts. Promising studies have shown that many phytochemicals such as curcumin, resveratrol and anthocyanins inhibit COX2 in cell cultures and animal models of colon and other cancers [9,10]. However, these preclinical findings are not necessarily translatable to humans, prompting concerns in the field. Below, we discuss some possible reasons and solutions along with human data from epidemiological and clinical trials that have used aspirin or phytochemicals to inhibit COX2. We also summarize the available human data on the synergism between aspirin and dietary phytochemicals in colon cancer prevention.

## 2. COX2 Inhibitors

According to current understanding, there are three COX isozymes: COX1, COX2, and COX3 [11,12,13]. COX1, which is constitutively expressed in most tissues, regulates renal blood flow and protects the integrity of the intestinal mucosa. COX2, which is highly inducible, is an important driver in tumor progression. COX3 is a splice variant of COX1. 

COX2 is usually absent, but this early response gene is transcriptionally upregulated by neoplastic and inflammatory stimuli (e.g., cytokines, growth factors, and mitogens). In colon cancer, COX2 is overexpressed in 40% to 50% of benign polyps and 80% to 90% of adenocarcinomas [12,14]. COX enzymes catalyze a key step in the conversion of arachidonate (AA) to prostaglandin H2 (PGH2), the immediate substrate for a series of cell-specific prostaglandin (PG) and thromboxane synthases. [14]. In particular, prostaglandin E2 (PGE2) is proinflammatory, and it promotes colon cancer through excessive lipid peroxidation and the formation of DNA adducts [15]. The suppressor 15-prostaglandin dehydrogenase (15-PGDH), which catalyzes the degradation of PGE2, is downregulated in colorectal adenoma and carcinoma cells [16]. 

The most effective way to inhibit COX2 clinically is with selective pharmacological inhibitors such as rofecoxib, valdecoxib, and celecoxib [9]. Celecoxib, when tested in patients with familial adenomatous polyposis who took 400 mg twice daily for six months significantly reduced the number and burden of colon polyps [17]. However, subsequent findings of severe cardiovascular risk associated with COX2 inhibitors in a small patient subpopulation caused rofecoxib and valdecoxib to be withdrawn from the market in 2004 and 2005, respectively [9]. 

Efforts to find more specific inhibitors of COX2 for long-term preventive use have not been successful except for aspirin that is taken in lower doses [9]. However, aspirin’s widespread use for preventing heart attacks and strokes has made it the most investigated NSAID for cancer prevention, particularly colorectal cancer [18]. Epidemiological studies have consistently observed an inverse association between aspirin use and risk of colorectal cancer [18]. A recent pooled analysis from four randomized, cardiovascular disease prevention trials of a long-term post-trial follow-up of nearly 14,000 patients showed that daily aspirin treatment for about five years associated with a 34% reduction in 20-year colorectal cancer mortality [1]. A separate meta-analysis from four randomized adenoma prevention trials of nearly 3000 patients with a history of colorectal adenoma or cancer showed that aspirin reduced the occurrence of advanced adenomas by 28% and aspirin reduced any adenoma by 17% [19]. Aspirin was also beneficial in patients with Lynch syndrome, an inherited disease with a high risk of colorectal cancer. In Lynch patients treated with aspirin for at least two years, there was a 50% or more reduction in the risk of colorectal cancer commencing five years after randomization and after aspirin had been discontinued [18]. A few observational studies have also found increased survival among colorectal cancer patients who use aspirin [18]. It should be noted that the chemopreventive activities of aspirin involve COX-independent mechanisms such as cyclin A2/CDK2 [20], phosphatidylinositol 3-kinase-related pathways (PIK3CA) [21], sirtuin 1 (SIRT1) [22], platelet inhibition [23], nuclear factor kappa B (NF-κB) [24], mammalian target of rapamycin (mTOR) signaling [25], DNA repair [26], etc. Readers are referred to comprehensive summaries on this topic in other reviews [26,27,28,29]. Although safe aspirin regimens can achieve only partial and transitory inhibition of COX2, it might be feasible to complement their cancer-protective benefits with agents such as phytochemicals that decrease COX2 expression or limit the bioactivity of COX2-derived PGE2 [30]. 

## 3. Phytochemicals as Natural Sources of Anti-COX2 Agents in Humans

Many phytochemicals—including flavonoids, phenolic acids, tannins, stilbenes, etc.—inhibit COX2 in cell cultures and animal models of colon cancer, and readers are referred to well-summarized reviews [9,10,11,31]. Here are only a few examples of these phytochemicals: curcumin, resveratrol, epigallocatechin gallate (EGCG), lycopene, anthocyanins, genistein, capsaicin, piperine, diosgenin, oleanolic acid, boswellic acid, lupeol, cineole, etc. Accordingly, only articles with results from human intervention trials are discussed here.

Curcumin is derived from turmeric (*Curcuma longa*), a golden spice that has been used for centuries in many Asian countries as part of the diet or as a coloring agent [9]. The anticancer and anti-COX2 effects of curcumin have been demonstrated in several cell culture and animal studies of cancers, including colorectal cancer [9]. One difficulty with curcumin treatment is low bioavailability, even when the supplement is taken in large doses [32]. A 2 g dose of curcumin resulted in undetectable serum curcumin levels in human patients [33]. In two clinical trials using an 8 g dose, plasma curcumin levels ranged from 22–41 ng/mL and 29–412 ng/mL [34,35], suggesting that absorption varies widely among individuals.

Although the systemic availability of curcumin is very low, 3600 mg curcumin administered orally each day for seven days accumulated in both normal and cancerous colorectal tissues from colon cancer patients [36]. The same study also found that curcumin did not affect COX2 protein levels in malignant colorectal tissue. In another study, neither curcumin nor its metabolites were detected in blood or urine, but curcumin was recovered from feces in patients with advanced colorectal cancer who are refractory to standard chemotherapies received *Curcuma* extract daily (36–180 mg) for up to four months, suggesting that it might be metabolized in the intestines and absorbed into colorectal tissues [37]. Accordingly, there have been efforts to modify curcumin such as by using implantable polymeric micelles or phospholipid-based delivery systems in an attempt to increase its accumulation, particularly in the gastrointestinal tract. Such approaches aim to use specially formulated curcumin to target COX2 more effectively, though that has yet to be determined in humans [38,39,40].

One study [41] investigated selective COX2 inhibition by an extract of *Pterocarpus marsupium* (the Indian kino tree), which contains pterostilbene, a stilbenoid also found in blueberries. In healthy human volunteers, oral use of 450 mg of *Pterocarpus marsupium* extract did not decrease PGE2 production. However, serum pterostilbene levels did increase, though they were five-fold lower than those observed when IC50 pterostilbene was used to inhibit PGE2 in LPS-stimulated human peripheral blood mononuclear cells (PBMC). This study strongly argues that a dose-finding study of *Pterocarpus marsupium* extract in humans is needed to validate the inhibition of PGE2 production observed in vitro. 

It is rather disappointing that phytochemicals with the potential to serve as natural low-toxicity COX2 inhibitors in cell cultures and animals have failed to inhibit COX2 in humans. Concerns and possible solutions relating to the effort to use dietary phytochemicals for colon cancer chemoprevention [42] are summarized below.

## 4. Is There a “Right” dose of Phytochemicals for Cancer Chemoprevention? 

Several lines of evidence suggest a nonlinear dose response for the protective effects of phytochemicals in humans. For example, the association between 25-hydroxyvitamin D, 25(OH)D levels and the risk of mortality in the general population was investigated in 13,331 nationally representative adults aged 20 years or older from mortality files linked to the Third National Health and Nutrition Examination Survey (NHANES III). Participants’ vitamin D levels were collected from 1988 through 1994, and the individuals were passively followed for mortality through 2000 [43]. There were 777 deaths from cardiovascular disease (CVD) and 424 deaths from all cancers; with 1806 deaths in total. Serum 25(OH)D levels below 17.8 ng/mL associated with a 26% increased rate of all-cause mortality whereas a population attributable risk was 3.1% [43]. However, U-shaped risk curves pointed out the possibility of increased risk when 25(OH)D levels were above 32.1 ng/mL [44]. Two cohort studies also obtained a U-shaped curve when 25(OH)D levels were plotted against colorectal or prostate cancer risk. With respect to colorectal cancer, blood samples taken in 1974 in Washington County, Maryland, from 25,620 volunteers were used to investigate the relationship of serum 25(OH)D with subsequent risk of developing colon cancer [45]. Between August 1975 and January 1983, 34 cases of colon cancer were matched to 67 controls by age, race, sex, and the month blood was taken. There was a 75% reduced risk of colon cancer in individuals with serum levels of 25(OH)D at 27−32 ng/mL, and it fell by 80% when levels were 33−41 ng/mL. However, the risk of colon cancer was not reduced in the group with 42−91 ng/mL serum 25(OH)D. In the case of prostate cancer, a longitudinal nested case-control study was conducted on Nordic men (from Norway, Finland, and Sweden), using banks of 200,000 serum samples [46]. Both low (≤19 nmol/L) and high (≥80 nmol/L) 25(OH)D serum concentrations associated with higher prostate cancer risk in a cohort of 622 prostate cancer cases and 1,451 matched controls. Subjects with a normal average serum concentration of 25(OH)D (40−60 nmol/L) had the lowest risk. The authors suspected that the reasons for U-shaped risk of prostate cancer might be due to similar 1,25-dihydroxyvitamin D(3) availability within the prostate because a low vitamin D serum concentration likely leads to a low tissue concentration and therefore weakened mitotic control of target cells. However, a high vitamin D level might lead to vitamin D resistance through increased inactivation as a result of enhanced expression of 24-hydroxylase [46]. Therefore, both high and low levels of blood vitamin D associate with higher prostate cancer risk. 

Thus, different studies have suggested that one should avoid vitamin D levels that are too low or too high. Moreover, findings from the Selenium and Vitamin E Cancer Prevention Trial (SELECT) further support the need to carefully select doses in chemoprevention clinical trials. Surprisingly, men with low selenium status were not beneficial from selenium supplementation, but men with high selenium status had increased risk of developing high-grade prostate cancer by selenium supplementation [47]. Moreover, vitamin E increased the risk of prostate cancer among men with low selenium status. Therefore, neither intervention was protective. This study suggests that men should avoid taking selenium or vitamin E supplementation at doses that go beyond recommended dietary intakes [47]. 

## 5. Less is More When Phytochemicals are Used for Cancer Chemoprevention 

To challenge the assumption that “more is better”, a study compared the pharmacokinetics and activity of a dietary dose of resveratrol with an intake 200 times higher (5 mg vs. 1 g) [48]. The dose–response relationship and metabolite profile of [(14)C]-resveratrol were first established in colorectal tissue collected from patients participating in a presurgical window trial. Importantly, the results proved that a dietary dose of resveratrol could reach colorectal mucosa, the target tissue, and therefore the full achievable concentration range in humans was defined. Importantly, patients who took 5 mg resveratrol daily had significantly higher levels of oxidative stress markers than a control population or patients who took 1 g daily, suggesting that the pro-oxidant effect of low-dose resveratrol was also evident in colorectal tissue of patients. The same article also reported a bell-shaped dose response to resveratrol in *Apc^min/+^* mice, a model of human colorectal cancer [48]. Taken together, these findings illustrate that low-level dietary exposure to resveratrol not only elicits biological changes in human tissues relevant to colorectal cancer prevention but is also more effective than high doses. It is possible that the same is true for other phytochemicals. Because multiple studies have reported nonlinear dose responses to phytochemicals in humans, it is crucial to identify the optimal dose range before conducting a human clinical trial. Robust pharmacodynamic biomarkers that correlate with the chemopreventive efficacy of phytochemicals at dose ranges that can be achieved in humans should be identified in preclinical models and ultimately tested in humans. 

## 6. Interactions Between Aspirin and High Fiber Diets in Colon Adenoma and Colon Cancer Incidence in Humans

The Polyp Prevention Trial (PPT) was a multicenter randomized clinical trial designed to evaluate the effects of a diet high in fiber (18 g/1000 kcal), high in fruits and vegetables (3.5 servings/1000 kcal), and low in fat (20% of total energy) on adenomatous polyp recurrence in the colon in both men and women (*n* = 2079) [49]. The results suggest that the dietary intervention did not influence the risk of recurrence of colorectal adenomas. In a subsequent analysis, use of NSAIDs and recurrence of colorectal adenomas in PPT was investigated [50]. Among the participants who completed the full follow-up (*n* = 1905), there was a significant reduction in overall adenoma recurrence among NSAID users, with the greatest effect seen with advanced polyps. Among aspirin users, there was a 40% reduction in the OR association of dose response for overall adenoma recurrence among those who ingested more than 325 mg per day but no change in the OR association among those took ≤325 mg per day. It is interesting, however, that several studies suggest that NSAIDS might modify the effects of calcium [51,52] and physical activity [53] on colon cancer risk. Accordingly, the analysis was performed to determine if NSAID use modified the effect of a low-fat, high-fiber diet on the recurrence of colorectal adenomas in PPT [49]. NSAIDs and aspirin significantly modified the association between the intervention and recurrence at baseline and throughout the trial (Table 1). The protective association observed for NSAID use was stronger among the controls than in the intervention group. The authors suggest that these results should be interpreted cautiously because the differences may have arisen by chance during the examination of multiple associations. Moreover, the participants in the PPT study were not randomly assigned to either dietary intervention or NSAID use. Importantly, however, this study suggests that a diet of low-fat, high-fiber rich in fruits and vegetables might lower the risk of recurrence of colorectal adenoma among individuals who do not regularly take NSAIDs [49].

In The Women’s Health Initiative Randomized Controlled Dietary Modification Trial, in order to motivate and support reductions in dietary fat, increase consumption of vegetables and fruits, and increase grain servings, an intensive behavioral modification program was used [54]. All participants were randomly assigned to two groups: the dietary intervention (*n* = 19,541) or a comparison group (*n* = 29,294) (Table 1). During a mean follow-up of 8.1 (SD, 1.7) years, there was a total of 480 cases of invasive colorectal cancer occurred. Primary analyses suggested that, by year 1, the reduction in the percentage of energy obtained from fat was 10.7% greater in the intervention group than in the comparison group. This difference between groups was mostly maintained (8.1% at year 6). The intervention group also made statistically significant increases in vegetable, fruit, and grain servings per day. However, despite these dietary changes, primary analyses suggested that there was no evidence that the intervention reduced the risk of invasive colorectal cancer during the follow-up period. Secondary analyses suggested that there were potential interactions with baseline aspirin use for that baseline high-dose aspirin (≥325 mg per day) users had a significantly decreased risk of developing invasive colorectal cancer versus non-users and low-dose aspirin (<325 mg per day) users. No interaction was observed with duration of aspirin use. After the 8.1 years of dietary modification, this study was extended to follow an additional 9.4 years of postintervention and the results were recently published in another article [55]. A total of 906 cases of colorectal cancer were identified during the intervention and postintervention periods. The colorectal cancer incidence was not different in the dietary modification group for any type of NSAID use, and none of the interactions with any category of NSAID use was statistically significant. However, there perhaps was modest evidence for an interaction (*p* = 0.07) with aspirin use at baseline, though the strength and duration of aspirin use at baseline did not alter the associations. Therefore, extended follow-up did not confirm the combined protective effects of aspirin and diet (reduction in dietary fat plus increased consumption of vegetables, fruits, and grains) on colorectal cancer risk among the postmenopausal women. The later report also did not confirm the initial findings that suggested a combined protective effect of aspirin and dietary modification. However, a high dose of aspirin (≥325 mg per day) is generally used to control specific symptoms such as fever, whereas a low dose or baby aspirin is protective against cancers, particularly colorectal cancer [18]. Additionally, it is conceivable that adherence to dietary modification declined over time during the intervention and might decline even more during the postintervention period. Thus, use of both low-dose aspirin and dietary modification by high-risk individuals who are susceptible to cancer may ease concerns about adherence. This approach may have a significant impact in that aspirin (600 mg per day) was not protective in hereditary colorectal cancer in patients with familial adenomatous polyposis (FAP) after more than one year of treatment [56], whereas a nine-month intervention with black raspberry suppositories, a rich source of salicylic acid and anthocyanins, associated with rectal polyp regression in FAP patients [57].

## 7. Conclusions

Both aspirin, and fruits and vegetables are attractive agents for colon cancer chemoprevention because they have little or no toxicity in humans. Salicylic acid, the principal metabolite of aspirin, is widely present in plant-derived foods, which further supports the possible synergistic effects of aspirin, and fruits and vegetables. The challenges of translating findings from preclinical models that demonstrate the protective effects of plant-derived phytochemicals to humans are starting to be addressed. Importantly, the doses tested in preclinical settings need to be relevant and achievable in humans. Furthermore, the bell/U-shaped response curves of phytochemicals suggest the need to identify specific pharmacodynamic biomarkers that correlate with the chemopreventive efficacy of phytochemicals or fruits and vegetables. It is conceivable that combining aspirin with fruits and vegetables is a promising chemopreventive approach, particularly in high-risk groups, because of its synergistic potential with little or no toxicity during years of intervention. 

## Reference

## Figures and Tables

**Table 1:** Interactions Between Aspirin and High Fiber Diets in Colon Adenoma and Colon Cancer Incidence in Humans

Trial Name	Primary end Point	Duration	Participants	Diet Intervention	Findings	Ref.
The Polyp Prevention Trial	Colorectal adenoma recurrence	Intervention for 3 years	1905 (intervention)	High-fiber (18 g/1000 kcal)	Interaction between intervention and aspirin (*p* = 0.03). Interaction between intervention and all NSAIDs (*p* = 0.008)	[48]
				High fruit and vegetable (3.5 servings/1000 kcal)		
				Low-fat (20% energy)		
The Women‘s Health Initiative Randomized Controlled Dietary Modification Trial	Invasive colorectal cancer incidence	Intervention for 8.1 years	19,541 (intervention) 29,294 (comparison)	Fruit and vegetable, at least 5 servings daily	Interaction between intervention and aspirin (*p* = 0.01)	[54]
				Grains, at least 6 servings daily		
				Fat: 20% energy		
The Women’s Health Initiative Randomized Controlled Dietary Modification Trial	Invasive colorectal cancer incidence	Intervention for 8.1 years and followed for an additional 9.4 years	19,541 (intervention) 29,294 (comparison)	Fruit and vegetable, at least 5 servings daily	Interaction between intervention and aspirin (*p* = 0.07). Interaction between intervention and all NSAIDs (*p* = 0.14)	[55]
				Grains, at least 6 servings daily		
				Fat: 20% energy		
